# Research on the establishment of evaluation index system of the aging construction of the community

**DOI:** 10.3389/fpubh.2023.1047625

**Published:** 2023-03-07

**Authors:** Kun Li, Wen-Bing Mei, Yi-Zhe Huang

**Affiliations:** ^1^School of Art and Design, Guangzhou University, Guangzhou, China; ^2^Department of Art Design, Guangdong Industry Polytechnic, Guangzhou, China

**Keywords:** age-appropriate community, index system, community home care, influencing factors, satisfaction

## Abstract

**Introduction:**

Constructing the evaluation index system for the community's aging-friendly construction can provide a decision-making basis for the overall investment and priority satisfaction strategy for the community's aging-friendly construction.

**Methods:**

Through questionnaire survey and statistical analysis, this paper evaluates and analyzes the satisfaction level of various indicators of the community's aging-friendly construction.

**Results:**

The results show that the community's humanistic care, public environment, and socio-economic criteria are significantly linearly linked to the overall construction satisfaction level. There are significant differences in the general satisfaction level of older adults in the variables of “age, educational background, residence duration, residence personnel, number of children, and marital status”.

**Discussion:**

To this end, the article concludes with specific recommendations that improving older-adult care services for older adults with low age and high education, helping older adults in the community to familiarize themselves with the community environment as soon as possible, cultivating the professional abilities of community service personnel, reflecting the warmth of the community for older-adult care and improving social security capabilities are the core content of the future community building for aging.

**- What do we already know about this topic**

At the beginning of community construction, less consideration was given to the pension problem of the elderly, resulting in the construction of the community and the pension needs of the elderly not matching. Therefore, how to build an index system for community aging construction, assess the construction status of community aging and propose improvement strategies is a topic of common concern of the government, industry and academia.

**- How does your research contribute to the field?**

Through the research and development method of combining quality and quantification, this paper constructs an index system for community aging construction under the community home care model, which can evaluate the construction status of community aging and put forward targeted improvement suggestions, so as to provide a quantifiable basis for improving the construction of community aging.

**- What are your research's implications toward theory, practice, or policy?**

Through the investigation and interview of the relevant groups of people in the community home care, this paper obtains the difference between the satisfaction and pressure of different types of elderly people on the indicators of community aging construction, which can not only provide effective supplements for the government to formulate community aging construction standards, but also provide a strategic basis for the investment of pension resources at the community level.

## 1. Introduction

The family planning policy implemented in the 1970s has led to a rapid decline in the birth rate of China's population. Coupled with improvements in the material living standards and health care conditions of the entire population, and a continued increase in the life expectancy of the elderly population, the number of elderly once again increases. Under the dual impact of the declining birth rate and prolonged life span of the elderly population, the function of family-based elderly care is weakening, while the pressure on social-based elderly care gradually increases. With the development of the economy and the acceleration of urbanization, industrialization, and globalization, it is becoming more common for the elderly to live separately from their children. In addition, the number of elderly living alone and empty-nesters is also increasing. Unlike foreign developed countries which enter the aging society gradually, China's aging is coming rapidly and presents the characteristics of “getting old before getting rich”. Simple elderly care institutions have the limitations of high costs, insufficient supply, and lack of warmth, which cannot meet the diversified needs of the elderly. Therefore, how to construct a elderly care model with Chinese characteristics and in line with Chinese aging habits has become a concern of all sectors of society (1).

With the rapid development of China's real estate industry, the community home-based care model has gradually emerged, and the Chinese government has also keenly observed this phenomenon. In 2005, it began to carry out community home-based care services with diversified investment subjects, public service objects, professional service teams, and diversified service methods as the objectives (2). After decades of development, China's urban communities have now established a comprehensive coverage of various forms of community home-based care service networks, constantly enriching community facilities, improving the life expectancy and quality of life of the elderly, and helping the Chinese elderly successfully age locally. Relies on the community as the carrier, the community home-based care model integrates various resources in the community, and organically combines family care and social care, which improves the limitations of monolithic and unprofessional family care services, and overcomes the defects of high cost and lack of warmth in institutional care. Community home-based care conforms to traditional Chinese living habits; it is a creative transformation of traditional elderly care culture and a new elderly care model that shines Chinese wisdom (3). We provide additional explanation for the concept of aged-oriented community. Many studies have shown that communities that are adapted to the needs of the elderly can facilitate their aging (4), but the space and field of communities vary, as do the needs of the elderly, so the concept of aged-oriented community needs to be interpreted in a specific context. To facilitate a summary for the concept of aged-oriented community, this paper follows Li (5) research and defines it as: within a certain community boundary, with a complete implementation of public services such as living facilities, medical care, shopping place, leisure facilities, and education services, which provides different social services for the elderly with different levels of health, so as to meet the diverse needs of different living subjects in the community. Official survey data shows that by 2017, more than 90% of the elderly have chosen community home-based care. It has developed into China's most crucial elderly care model, widely recognized in society.

Through recent years' exploration and practice of the home-based elderly care model in urban communities in China, the social care problem for the elderly has been more effectively resolved. As an essential element of elderly protection, the ultimate goal of developing community home-based care services is to improve the quality of life and life satisfaction of the elderly. Among them, whether the construction of a suitable age-oriented community can match the needs of the elderly, and whether the diversified needs of the elderly can be effectively met in the community are the core issues and critical links that affect the development of the community's home-based care model and the realization of its functions (6). Therefore, under the community home-based care model, identifying the satisfaction degree of the elderly with various elements of the community's aging construction and adopting corresponding adjustment strategies has become an important issue facing and urgently needs to be resolved in the development of China's current social elderly care service industry. In summary, the research objectives are as follows:

1. Through questionnaire surveys, this paper explores the construction indicators of aged-oriented community construction. It aims to establish an evaluation index system of the aging construction of the community. Cooperating with the promotion of relevant policies, it develops a comprehensive community home-based care model.
2. Through statistical analyses, the study discusses the community's aging construction satisfaction degree and importance degree to the community's home-based elderly. It will help guide the community's home-based care resources investment at the overall level and formulate priority satisfaction strategies.

## 2. Literature review

### 2.1. Research on the connotation and characteristics of community home-based care

China's traditional caring model are broadly divided into three types: home-based care, community-based care and institutional care (7). Existing studies have distinguished among the three from two perspectives: in terms of service location, home-based care is mainly located within the family; community-based care is mainly located in community institutions, such as day care centers; and institutional care is mainly located in specialized nursing homes. In terms of service target audience, home-based care mainly covers healthy elderly who are capable of taking care of themselves; community-based care mainly targets elderly people who can partially take care of themselves, providing them with community-level care; and institutional care provides professional care for elderly people who are not capable of taking care of themselves.

Although “home-based care” and “community-based care” exist as independent models in the above division, they are closely related in the service boundary and actual operation (8). In the last decade or so, as China's economy grows, the functionality of community has become stronger and stronger, and it has become more and more popular for the elderly to stay at home and receive community caring service, thus giving rise to the new “community home-based care model”. In terms of connotation, community home-based care is not a simple combination of home-based and community caring service, but rather emphasizes the community's “support” function and the home-based care's radiation effect.

Therefore, this study defines the community home-based care model as “a model that provides community elderly care service to the elderly at home, through the community's integration of the society's elderly care resources, with the family as the core, the community as the backbone, and specialized service institutions as the carrier, combining home and community institutions.

The concept of community-based care for the elderly originated from British community care. In the 1960s, the United Kingdom began to develop community care. After more than 20 years of development, it has matured and become increasingly perfect, and was later recognized and imitated by various countries (9) The term “community home-based care” was first mentioned in the document “Constructing a Community Home-based Care System” issued by China in 2001, which subsequently aroused heated discussions and extensive practices in the industry and academia (10). Community home-based care is the combination of family and social care, that is, “home-based care in the community”. It uses the family as the place for care and the community as a resource platform to divert social care resources to the elderly. This elderly care model emphasizes the role of the community in integrating the social elderly care resources in the operation of the elderly care model (11). From the current research situation, the concept of “community home-based care” mainly includes three aspects. The first is family nature. In community home-based care, the elderly choose their own homes as a place for care rather than go to specialized institutions for centralized care. Community home-based care for the elderly is an optimization of traditional family care, reflecting the field-type difference from social institutions (12). The second is community. The community home-based care model emphasizes the integration of “community” to social elderly care resources. By coordinating the relationship between society, community and family, the community allocates and integrates the resources of traditional family-based elderly care, professional institution elderly care, and social security pension. On the one hand, professional elderly care institutions or social security departments can provide more convenient, professional and diversified elderly care services to the elderly at home through the community. On the other hand, the elderly at home can also connect with the social security department or professional elderly care institutions through the community to meet their own multi-level and unique elderly care needs (13). The third is sociality. Community home-based care is a compound type of care that highlights family care, supplemented by institutional care. It is an organic combination of family care and community care. On the one hand, the community home-based care model strengthens the positive impacts of family care's environmental, material, and spiritual elements on the elderly. On the other hand, it introduces professional services, nursing care, and medical consolation from social care institutions to the elderly to increase the function of home-based care (14).

### 2.2. Construction of indicators and evaluation systems related to aged-oriented communities

Existing research on the construction of community aging around the community home level is relatively limited, while the research on the overall demand for elderly care services is relatively high. The WHO issued the “International Consensus on Establishing Long-term Care Policies for the Elderly” in 2000, which set independence, participation, care, self-enrichment and dignity as the goals of long-term care policies for the elderly (15). In 2002, the “International Program of Action on Aging Issues” promulgated by the Vienna International Convention Organization proposed to focus on the social, economic and human rights development of the elderly. In April of the same year, WHO presented the “Active aging Policy Framework” report at the United Nations Second World Conference on Aging. The report defined active aging as providing the best possible opportunity for health, participation, and protection to improve the quality of life. “Active” means that the elderly must participate in social, economic, cultural, spiritual and public welfare affairs in addition to being in good health and being able to participate in physical activities and the workforce (16). Accessing the elderly's needs from the five dimensions of social resources, economic resources, mental health, physical health, and daily activities (ADL), the “Old Americans Resources and Service (OARS)” developed by Duke University in the United States suggested 24 indicators include housekeeping, transportation, nursing, care, nursing, and mental health (17). Forder summarizes the needs of the elderly into four aspects: medical health, economic needs, living environment, and social psychology (18). The “Five Lao-You” elderly care system proposed by Chinese academic circles since 1982, namely, “the old have security, the old have medical care, the old have learning resources, the old can achieve something, and the old have fun” is the Chinese aging policy and the earliest classification standard used in the research field (19). With the progress of the times and the development of society, especially the continuous improvement of the social pension security system, the socio-economic elderly care system developed based on the “Five Lao-You”, which is in line with China's national conditions, has become a common and recognized method of classification in the academic circles (20). It can be seen that the elderly's needs for aging care have gradually changed from “individual” to “social”, from “physical” to “spiritual”, and from “survivability” to “development”, which has been universally recognized by the international community.

### 2.3. Research on the influencing factors and satisfaction degree of elderly care demand

In order to measure the status of active aging in various regions, the European Commission (EC) and the United Nations Economic Commission (UNECE/EC) for Europe introduced the Active Aging Indicator System (AAI) in 2012, and formulated relevant policies to promote active aging in various countries and track its development. The AAI is a multi-dimensional index that measures the independent standards of living, participation in the labor market, and social activities of the elderly over 55, and their ability to age actively. AAI provides a social perspective on the aging phenomenon and is a helpful tool for top-down policy design (21). Liu and Yang (22) used the CHARLS and CGSS databases, borrowed from the EU's active aging measurement framework, and used the combination of AHP and DEA to design the China Active Aging Index (CAI) and measured the indicators of active aging in China's three major regions and 28 provinces. The result shows that the development level of active aging in China is unbalanced, with a trend of high in the east and low in the west. The level of active aging in urban and rural areas is also significantly different, with the level of the male generally higher than that of the female, and the gap is gradually widened with age (22). Costa proposed the “Competence-Press Model” (CPM), an ecological model about the elderly and their environmental adaptation, and calculated the linear functional relationship between age, degree of physiological function decline, family structure, living mode, financial ability, and other differential variables and the environmental adaptation ability of the elderly (23). Guo and Hao (24) pointed out that the elderly's satisfaction with medical and spiritual levels is affected by factors such as age, number of children, people living with them and health status (24).

To sum up, there is no uniform standard for classifying the types of elderly care needs in academic circles. Based on different perspectives, the types of elderly care needs are not the same, which also fully illustrates the complexity and diversification of the construction of aging communities. Similarly, for a long time, though domestic and foreign scholars have conducted extensive investigations and studies on the influencing factors and satisfaction issues of elderly care needs, the existing research is limited to the elderly physiology, psychological and economic resources. In contrast, less attention has been paid to the construction of aging communities. Therefore, the existing research conclusions do not strongly support methods of improving the community's aging construction. This paper intends to start from the indicator system of community aging construction under the community home-based care model, through a combination of questionnaire surveys and statistical analysis, explore the community's aging constructions' satisfaction degree and influencing factors from the community home-based elderly, thus constructing a sustainable community home-based elderly care model.

## 3. Materials and methods

According to the previous research results of this research team, the indicators of a community's aging construction under the community home-based care model are divided into four criteria: human care, public environment, health care, and social economy, and 48 indicators. The specific contents are shown in Table 1 (20). The purpose of this research is to explore the elderly's overall satisfaction with the community's aging construction, and to analyze the influencing factors, so as to build an evaluation index system of the aging construction of the community. This study aims to construct an evaluation index system of the aging construction of the community based on the fact that professional practitioners have rich practical experience, and the elderly in the community can present detailed care needs and Influencing factors of community aging satisfaction (25). Therefore, this study uses questionnaire survey to conduct extensive questionnaire research on the home-based elderly and related professional groups, and uses exploratory factor analysis to construct the evaluation index system of the aging construction of the community. Using multiple regression analysis to explore the influence of the variables of community aging construction on the overall satisfaction of community aging construction. The research was conducted in two stages. The first is a questionnaire survey, which assesses the overall satisfaction degree of the community, and the satisfaction and importance of each index for the elderly with community home-based care. In this way, the construction level of community aged-oriented satisfaction was measured to provide decision-making reference for relevant departments to optimize community aging construction. The second stage is statistical analysis. Through statistical analysis of the questionnaire data, it is expected to analyze the influencing factors of community seniors' satisfaction with the community's aging construction, so as to optimize the connotation of and provide empirical support for the community's aging construction.

**Table 1:** Community's aging construction indicators.

**Indicator criteria**	**Indicator name**
A. Humanistic Care	A1.Organizating community activity, A2.Exchange community information, A3.Improving community services, A4.Providing spiritual consolation A5.Improving neighborhood relations, A6.Providing psychological guidance, A7.Foster community identity, A8.Providing cultural education, A9.Organizing cultural shows, A10.Handling related affairs, A11.Publicity policy, A12. Managing elderly supplies, A13. Providing legal aid
B. Public environment	B1.Improving fitness facilities, B2.Providing leisure seating, B3.Improving lighting facilities, B4.Improving sanitation facilities, B5.Road accessibility, B6.Managing community vehicles, B7.Improving community roads, B8.Improving physical environment B9.Building facilities for the elderly, B10.Activity space with dynamic separation, B11.Formulate safety measures, B12.Beautify community environment, B13.Ensure environmental space accessibility, B14.Supporting living facilities, B15.Ensure public transportation
C. Health care	C1.Providing healthcare knowledge talks, C2.Providing rehabilitation nursing guidance, C3.Providing regular physical examination, C4.Chronic diseases check, C5.Setting up an emergency rescue system, C6.Supporting community clinic, C7.Building community day care centers, C8.Providing drug dispensing and delivery services, C9.Providing home-visit medical services, C10.Providing accompanying medical services
D. Social economy	D1.Building elderly canteen, D2.Providing housekeeping services, D3.Providing life care services, D4.Providing social security services, D5.Public service information content, D6.Providing intermediary service consultation, D7.Establishing elderly information files, D8.Building safety monitoring system, D9.Providing internet elderly care information, D10.Providing online clinic service

### 3.1. The first stage: Questionnaire survey

#### 3.1.1. Development of the questionnaire

The questionnaire design contains two parts: basic socio-economic information and content items. The basic socio-economic information includes gender, age, education level, marital status, monthly income, occupation, age in the community, health status, length of residence, number of residents, number of children, community style, and the community's location. Content items are based on the four criteria and 48 sub-indexes obtained by the research team's previous research results. The study uses the 5-point Likert Scale (5-point Likert Scale) to measure the satisfaction and importance level of each sub-indexes, which descends from the highest 5 “very satisfied/important” to the lowest 1 “very dissatisfied/ not very important”.

#### 3.1.2. Questionnaire object

Since this phase of the test involves the professional construction standards of community age-oriented care under the community home-based care model, the test subjects must have relevant experience in community home-based care and be able to identify the importance of the construction indicators. In addition, the current technological advancement has brought about tremendous changes in society, so the construction of a community suitable for aging must be forward-looking. Therefore, in this study, elderly people over 60 years old who choose community home-based care mode and “quasi-elderly” people over 50 years old who will become elderly people in the next 10 years were selected as the target subjects, and the questionnaire was conducted online using “Wechat Questionnaire Star”.

#### 3.1.3. Questionnaire expert review

The indicators in this study have been verified by previous literature studies, and the content of its measurement tool is sufficient to cover the topics to be discussed. In addition, before the formal testing of our questionnaire, we also discussed with experts and scholars in related fields to ensure the validity and reliability of the questionnaire.

#### 3.1.4. Questionnaire implementation

The questionnaire survey lasts for 5 months and the distribution time is from May 17th to October 17th, 2020. Since most of the test subjects are elderly people and are limited by network technology and multimedia equipment, the research team has organized 40 members to track and serve the test subjects to ensure that the test subjects can fully understand the meaning of the questionnaire items and the test so that the assessment of the importance of construction indicators can be accurately reflected in the questionnaire.

### 3.2. The second stage: Statistical analysis

#### 3.2.1. Analysis of sample quantity

The questionnaire survey in this study lasted for 5 months, and the final number of questionnaires returned was 605. Because researchers tracked and served the test subjects, the quality of the questionnaires filled out was high. After deleting the unqualified and invalid questionnaires, 576 valid questionnaires were actually obtained, and the statistical effective rate reached 95.2%. The major content items of the questionnaire contains 48 items, and the sample size has reached 5 times of the questionnaire items (26). The minimum sample size for studying human behavior is 200, and the absolute sample size is appropriate for factor analysis (27).

#### 3.2.2. Sample structure analysis

The statistical results that basic information show that 46.88% of the subjects are men and 53.13% are women. The elderly over 60 accounted for 78.3%; 72.4% had a spouse; 57.12% were fully healthy; 55.56% of the respondents lived in commercial housing; 40.45% of the respondents lived in the old town area.

#### 3.2.3. Appropriateness and reliability analysis of questionnaire results

In this study, SPSS statistical software was used for data analysis. After reliability analysis and detection, the Cronbach's α value was 0.981, which is a high level of reliability, indicating that the reliability, consistency and stability of all related item data measured in this study have reached academic requirements, suitable for factor analysis (28).

## 4. Results and discussion

### 4.1. Factor analysis extraction criteria and nomenclature

#### 4.1.1. Factor analysis and nomenclature of the “humanistic care” level

According to the previous research, the “humanistic care” level of the community's aging construction includes 13 indicators “A1. Organizing community activity, A2. Exchange community information, A3. Improving community services, A4. Providing spiritual consolation A5. Improving neighborhood relations, A6. Providing psychological guidance, A7. Foster community identity, A8. Providing cultural education, A9. Organizing cultural shows, A10. Handling related affairs, A11.Publicity policy, A12. Managing elderly supplies, A13. Providing legal aid”. The KMO (Kaiser-Meyer-Olkin) value is 0.944 after the covariation relationship test. The Bartlett spherical test shows the approximate chi-square is 6803.659, the *p*-value is 0.000. The values of KMO and the Bartlett spherical test indicate that all relevant indicators of the “humanistic care” level have good internal consistency and reach a significant level suitable for factor analysis (29).

Two factors are extracted from 13 indicators. The Cronbach's α coefficient of factor 1 is 0.938, the Eigenvalue is 5.168, and the variance extracted value is 39.754%; the Cronbach's α coefficient of factor 2 is 0.934, the Eigenvalue is 4.630, the variance extracted value is 35.613%, and the total amount of variance extracted is 75.367%. See Table 2 for details.

**Table 2:** Factor analysis result of “humanistic care” dimension.

**Construction index**	**Community care**	**Social care**
A2. Exchange community information	0.886	
A1. Organizing community activity	0.858	
A5. Improving neighborhood relations	0.793	
A9. Organizing cultural shows	0.758	
A7. Foster community identity	0.739	
A3. Improving community services	0.715	
A4. Providing spiritual consolation	0.636	
A8. Providing cultural education	0.622	
A11. Publicity policy		0.891
A13. Providing legal aid		0.868
A12. Managing elderly supplies		0.863
A10. Handling related affairs		0.835
A6. Providing psychological guidance		0.653
Cronbach's α	0.938	0.934
Cumulative Cronbach's α	0.952	
Eigenvalues	5.168	4.630
Variance contribution(%)	39.754	35.613
Cumulative variance contribution value(%)	39.754	75.367
KMO	0.944	

It can be seen from Table 2 that the extracted factor 1 contains 8 construction indicators, a comprehensive analysis of these eight indicators is related to a care project that can be provided to the elderly within the community's scope. Thus, this study named factor 1 “community care”. The extracted factor 2 contains 5 indicators, a comprehensive analysis of these five indicators is related to a care project that requires the help of a community platform to introduce social resources into the community to provide for the elderly in the community. Thus, this study named factor 2 as “social care”.

#### 4.1.2. Factor analysis and nomenclature of the “public environment” level

According to the previous research, the aging construction of communities at the level of “public environment” includes 15 indicators “B1. Improving fitness facilities, B2. Providing leisure seating, B3. Improving lighting facilities, B4. Improving sanitation facilities, B5. Road accessibility, B6. Managing community vehicles, B7. Improving community roads, B8. Improving physical environment B9. Building facilities for the elderly, B10. Activity space with dynamic separation, B11. Formulate safety measures, B12. Beautify community environment, B13. Ensure environmental space accessibility, B14. Supporting living facilities, B15. Ensure public transportation” 15 indicators. The KMO value is 0.974 after the covariation relationship test. The Bartlett spherical test shows the approximate chi-square is 7231.464, the *p-*value is 0.000.

Three factors are extracted from 15 indicators. The Cronbach's α coefficient of factor 1 is 0.941, the Eigenvalue is 5.445, and the variance extracted value is 36.303%; the Cronbach's α coefficient of factor 2 is 0.903, the Eigenvalue is 3.366, the variance extracted value is 22.442%, and the Cronbach's α coefficient of factor 3 is 0.865, The Eigenvalue is 2.470, the variance extracted value is 16.468%, and the total of variance extracted value is 75.213%. See Table 3 for details.

**Table 3:** Factor analysis result of “public environment” dimension.

**Construction index**	**Physical environment**	**Facility environment**	**Supporting environment**
B8. Improving physical environment	0.811		
B7. Improving community roads	0.810		
B6. Managing community vehicles	0.795		
B12. Beautify community environment	0.731		
B10. Activity space with dynamic separation	0.731		
B13. Ensure environmental space accessibility	0.704		
B11. Formulate safety measures	0.692		
B9. Building facilities for the elderly	0.621		
B5. Road accessibility		0.534	
B2. Providing leisure seating		0.804	
B3. Improving lighting facilities		0.800	
B4. Improving sanitation facilities		0.714	
B1. Improving fitness facilities		0.628	
B15. Ensure public transportation			0.855
B14. Supporting living facilities			0.843
Cronbach's α	0.941	0.903	0.865
Cumulative Cronbach's α	0.954		
Eigenvalues	5.445	3.366	2.470
Variance contribution(%)	36.303	22.442	16.468
Cumulative variance contribution value(%)	36.303	58.745	75.213
KMO	0.947		

It can be seen from Table 3 that the extracted factor 1 contains 8 indicators, a comprehensive analysis of these eight indicators relates to the physical environment of the community's home for the elderly. Thus, this study named factor 1 as “physical environment”. The extracted factor 2 contains 5 indicators, a comprehensive analysis of these five indicators is related to the elderly care facilities provided for the elderly in the community. Thus, this study named factor 2 as “facility environment”. The extracted factor 3 contains 2 indicators, a comprehensive analysis of these two indicators is related to providing supporting functions for the society to meet the needs of the elderly in the community. Thus, this study named factor 3 as “supporting environment”.

#### 4.1.3. Factor analysis and nomenclature of the “health care” level

According to the previous research, the “health care” level of the community's aging construction includes ten indicators” C1. Providing healthcare knowledge talks, C2. Providing rehabilitation nursing guidance, C3. Providing regular physical examination, C4. Chronic diseases check, C5. Setting up an emergency rescue system, C6. Supporting community clinic, C7. Building community day care centers, C8. Providing drug dispensing and delivery services, C9. Providing home-visit medical services, C10. Providing accompanying medical services”. The KMO value is 0.926 after the covariation relationship test. The Bartlett spherical test shows the approximate chi-square is 6136.266, the *p*-value is 0.000.

Two factors are extracted from 10 indicators. The Cronbach's α coefficient of factor 1 is 0.951, the Eigenvalue is 5.028, and the variance extracted value is 50.279%; the Cronbach's α coefficient of factor 2 is 0.845, the Eigenvalue is 2.890, the variance extracted value is 28.901%, and the total of variance extracted value is 79.180%. See Table 4 for details.

**Table 4:** Factor analysis result of “health care” dimension.

**Construction index**	**Health management**	**Medical visits**
C4. Chronic diseases check	0.880	
C9. Providing home-visit medical services	0.830	
C10.Providing accompanying medical services	0.823	
C3. Providing regular physical examination	0.810	
C8. Providing drug dispensing and delivery service	0.791	
C2. Providing rehabilitation nursing guidance	0.701	
C5. Setting up an emergency rescue system	0.641	
C6. Supporting community clinic		0.924
C7. Building community day care centers		0.668
C1. Providing healthcare knowledge talks		0.612
Cronbach's α	0.951	0.845
Cumulative Cronbach's α	0.956	
Eigenvalues	5.028	2.890
Variance contribution(%)	50.279	28.901
Cumulative variance contribution value(%)	50.279	79.180
KMO	0.926	

It can be seen from Table 4 that the extracted factor 1 contains 7 indicators, a comprehensive analysis of these seven indicators is related to those elderly people living at home in the community providing preventive or rehabilitative elderly care services. Thus, this study named factor 1 as “health management”. The extracted factor 2 contains 3 indicators, a comprehensive analysis of these three indicators is related to all belonging to the needs of providing supplementary medical services for the elderly in the community. Thus, this study named factor 2 as “medical visits.”

#### 4.1.4. Factor analysis and naming of the “social economy” level

According to the previous research, the “social economy” level of the community's aging construction includes ten indicators: “D1. Building elderly canteen, D2. Providing housekeeping services, D3. Providing life care services, D4. Providing social security services, D5. Public service information content, D6. Providing intermediary service consultation, D7. Establishing elderly information files, D8. Building safety monitoring system, D9. Providing internet elderly care information, D10. Providing online clinic service”. The KMO value is 0.929 after the covariation relationship test. The Bartlett spherical test shows the approximate chi-square is 6983.130, the *p*-value is 0.000.

Two factors are extracted from 10 indicators. The Cronbach's α coefficient of factor 1 is 0.970, the Eigenvalue is 6.087, and the variance extracted value is 60.871%; the Cronbach's α coefficient of factor 2 is 0.864, the Eigenvalue is 2.427, the variance extracted value is 24.268%, and the total amount of variance extracted value is 85.139%. See Table 5 for details.

**Table 5:** Factor analysis result of “social economy” dimension.

**Construction index**	**Public security**	**Daily life**
D5. Public service information content	0.913	
D6. Providing intermediary service consultation	0.901	
D7. Establishing elderly information files	0.900	
D9. Providing internet elderly care information	0.881	
D10. Providing online clinic service	0.877	
D8. Building safety monitoring system	0.845	
D4. Providing social security services	0.833	
D1. Building elderly canteen		0.930
D2. Providing housekeeping services		0.816
D3. Providing life care services		0.593
Cronbach's α	0.970	0.864
Cumulative Cronbach's α	0.959	
Eigenvalues	6.087	2.427
Variance contribution(%)	60.871	24.268
Cumulative variance contribution value(%)	60.871	85.139
KMO	0.929	

It can be seen from Table 5 that the extracted factor 1 contains 7 indicators, a comprehensive analysis of these seven indicators is related to those elderly living at home in the community providing public security services. Thus, this study named factor 1 as “public security”. The extracted factor 2 contains 3 indicators, a comprehensive analysis of these three indicators is related to the need to provide elderly care services for the elderly in the community. Thus, this study named factor 2 as “daily life”.

### 4.2. The influence of basic structural variables on the satisfaction evaluation of the community's aging construction index

This study uses a One-way analysis of variance and an independent sample *t*-test to detect whether there are significant differences between the various dimensions of the community's aging construction indicators and the overall satisfaction of the community's aging construction with different basic variables. The basic variables detected by the One-way analysis of variance include age, education level, monthly income, occupation, community age, health status, community residence time, residents, number of children, community style, and community location. The Significant test results are shown in Table 6.

**Table 6:** Investigator's structural variables analysis table.

**Variable definition**	**Variable**	***f***	***p***	**Scheffe'**
The overall satisfaction of the aging community	Age	9.149	0.000	(over 71 years / 61–70 years)>Under 60
	Education	5.826	0.001	Below university>University and above
	Length of residence	3.540	0.030	5–10 years>Within 5 years
	Cohabitants	4.084	0.007	(Spouse/ children)>housemaid
	Number of children	6.119	0.002	(Multiple children/ 1 child)>No children
	Marital status	2.036	0.043	Married> No spouse

^*^p < 0.05, ^**^p < 0.01, ^***^p < 0.005.

It can be seen from Table 6 that there is a significant difference in the overall satisfaction in the community's age-oriented structure. The results show that people over 71 years and 61–70 years old have higher satisfaction levels than those under 60; There are significant differences in the evaluation of the education of the senior community in the academic structure. The results show that people with a university degree or less have higher satisfaction than those with a college degree or above. Significant differences exist in the overall satisfaction assessment of the length of residence in the community's aging construction status. The results show that people who lived for 5–10 years have higher satisfaction levels than those within 5 years. There is a significant difference in the community's overall satisfaction with the cohabitant structure. Test the results show that cohabitants with spouses or children have higher satisfaction levels than cohabitants with a housemaid. There is a significant difference in the overall satisfaction of the number of children in the community. The results show that seniors with more than one child are more satisfied than those without children. There is a significant difference in the overall satisfaction of the number of Marital statuses in the community. The results show that seniors with a spouse are more satisfied than those without a spouse.

### 4.3. The impact of criteria variables on the overall satisfaction evaluation of the community's aging construction

This study uses Multiple Regression Analysis to explore whether there is a linear relationship between the variables of various dimensions and the overall satisfaction evaluation of the community's aging construction, and establish a regression model. In the regression model, the dependent variable (y) is the overall satisfaction of the community's aging construction, and the independent variable (x_n_) is x_1_ humanistic care, x_2_ public environment, x_3_ health care, and x_4_ social economy. The test results are shown in Table 7.

**Table 7:** Analysis of the overall satisfaction degree of the construction in the aging community.

**Model**	**Standardized coefficient**	***t*-test**	***p***	**Collinearity statistics**		**Adjusted R^2^**	**Durbin-Watson**
	**Beta**			**Tolerance**	**VIF**		
1 (constant)		16.231	0.000			0.274	1.886
x_1_	0.525	14.780	0.000	1.000	1.000		
2 (constant)		10.778	0.000			0.294		
x_1_	0.401	8.675	0.000	0.575	1.739		
x_2_	0.191	4.124	0.000	0.575	1.729		
3 (constant)		11.112	0.000			0.305		
x_1_	0.455	9.034	0.000	0.478	2.091		
x_2_	0.209	4.498	0.000	0.562	1.780		
x_4_	−0.114	−2.636	0.009	0.645	1.550		

y: The overall satisfaction of the aging community, ^*^p < 0.05, ^**^p < 0.01, ^***^p < 0.005.

From Table 7, Through multiple regression analysis, the overall satisfaction of the community's aging construction (y) and humanistic care (x_1_), public environment (x_2_) and social economy (x_4_) indeed has a significant linear relationship. In this case, the regression analysis resulted in three models, of which model three had the largest Adjusted R^2^ = 0.305. From model three, we noticed that the regression coefficients of all independent variables are significant. This also indicates that human care (x_1_), public environment (x_2_), and social economy (x_4_) have a significant influence on the overall satisfaction of the community's aging construction (y) (30, 31). Therefore, the regression model is established as formula (1):


$$y=0.455\times x_{1}+0.209\times x_{2}-0.114\times x_{4}$$

This model has a resolving power of 0.305, which has reached the level value (0.3) required for a general academic paper (32), so the regression model coalesces well. Through residual analysis, the Durbin-Watson value of the regression model is 1.886, which is close to 2. According to collinearity statistics, the tolerance values of the dimensions are all >0.1, and the VIF is <10. Therefore, the regression model's residuals comply with the assumption of normality, constancy, and independence. The collinearity problem does not exist (33)^.^ Therefore, the regression model constructed in this study possesses reasonableness, high explanatory power, and is credible.

## 5. Conclusions and suggestions

In the era when the whole society is paying attention to the development of the elderly care service industry, the community home-based care model has gradually entered a standardized and mature state. Although the relevant norms and standards in China are constantly updated, and the community home-based care service system is constantly improved, perfecting the construction level of appropriate aging in the community is the topic that the elderly and related staff are keen on and concerned about. Suppose we can build a complete and precise community aging construction indicator system when working at the front end, and identify the community's elders' satisfaction with community aging construction. In that case, it will surely assist the government authorities and community home-based care workers in finding suitable methods that accommodate the needs of the elderly in the community. The conclusions and suggestions of this study are described as follows:

### 5.1. Research conclusion

#### 5.1.1. Construction of the evaluation index system of the community

**Figure 1:**
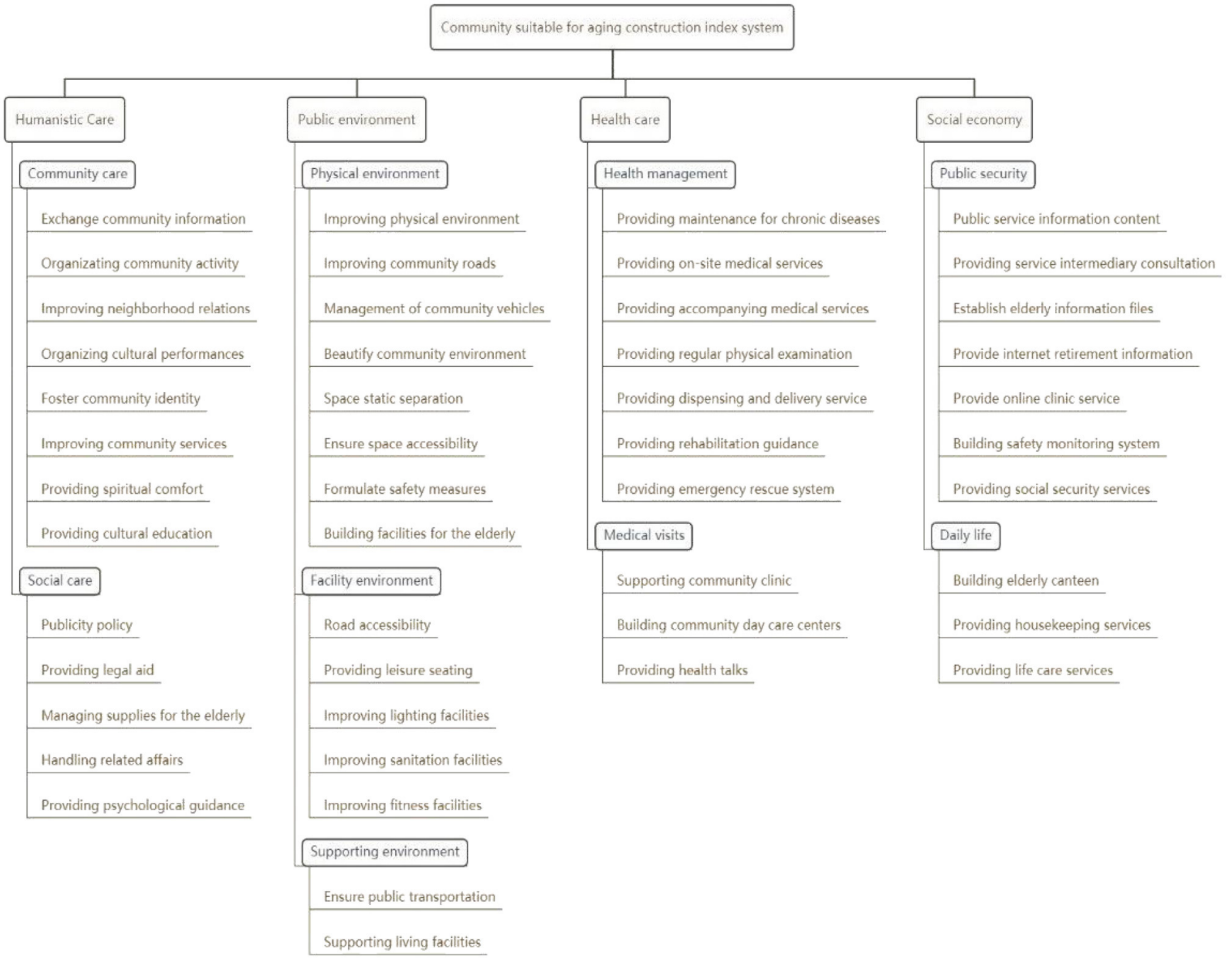
Evaluation index system of the aging construction of community.

This study used factor analysis to extract and name the dimensions of the community's aging construction indicators under the community home-based care model, and finally generated four criteria: human care, public environment, health care, and social economy. It is a system composed of four aspects, nine factors, and 48 construction indicators, as shown in Figure 1.

#### 5.1.2. The construction of community suitable for aging should be synchronized with social and economic development

In this study, it can be found from the differences in the satisfaction level from the respondents with different age and education background that the construction of community suitable for aging has fallen behind the speed of social and economic development.

The “Age” variable shows a significant difference between over 71 years old/ 61–70 years old and under 60 years old” in the overall satisfaction evaluation of the community's appropriate aging. It reflects that the construction of community aging needs to keep pace with the times and meet the needs of the elderly in the future (34). The “Educational” variable shows a significant difference between Below university and University and above in evaluating the overall satisfaction of the senior community. This is because low-educated persons are generally restricted by many factors such as knowledge structure and their own insights. The information and high-level experience obtained from the outside world in the process of individual growth will be lower than that of high-educated persons, so the demand for elderly care services is significantly lower for those with higher education (35). However, with the development of the economy and the general increase in the population with higher education, how to improve the level of community aging construction to meet the needs of senior citizens with high education will be the focus of future community aging construction.

#### 5.1.3. The construction of community aging should improve the quality of community elderly care services

The “Length of residence” variable shows a significant difference between 5 and 10 years and within 5 years in assessing the overall satisfaction of the senior community. Therefore, how to help the elderly in the community quickly integrate into community life and improve their familiarity with the community is the focus of the work of community staff in the future community home-based care model (36). The variable of “Cohabitants” shows a significant difference between spouse/children and housemaids in the overall satisfaction evaluation of the community's senior fitness. This is because the people who choose to live with helpers or service personnel are usually elderly or elderly with defective life governance capabilities. This part of the elderly usually has a higher demand for community care services than the ordinary elderly (37). This also reflects that, on the one hand, there is still a big gap between the current construction content of the age-oriented community and the needs of the elderly or the elderly with poor living governance ability. It is one of the problems to be solved in future community aging construction. On the other hand, it also shows that the nanny or the professional nursing level of service personnel urgently needs to be improved to compensate for the age-oriented community's insufficiency. The “number of children” variable shows a significant difference between multiple children/1 child and no children in the overall satisfaction evaluation of the community's aging. The “marital status” variable shows a significant difference between spouse and no spouse in assessing the overall satisfaction of aging in the community. This is because widowed elderly without children or spouses need to obtain more elderly care needs from the community when living at home than those with children or spouses. It also reflects the lack of warmth in the current community's aging construction. Therefore, how to let the lonely elderly or empty-nester in the community realize the warmth of the family is an urgent problem to be solved in the construction of suitable aging communities in the future (38).

#### 5.1.4. Humanistic care is the core element of community aging construction

After performing multiple regression analysis, this study constructed a linear regression model of the overall community's aging construction satisfaction. It can be seen from the formula (1) that the linear relationship between the “Humanistic care” dimension and the overall satisfaction of the community's aging construction is the strongest (β = 0.455), followed by the “Public environment” dimension (β = 0.209) and the “Social economy” dimension (β = −0.114). Among them, the overall satisfaction of the community's aging construction has a positive linear relationship with the two dimensions of humanistic care and public environment, which shows that humanistic care and public environment are the core content of the community's aging construction under the community home-based care. With the development of China's economy and the rise of the real estate industry, especially the implementation of the old town area renovation policy, the public environment of the community has been generally improved, and the elderly's needs in the public spaces are effectively met. However, undeniably, the construction of China's spiritual civilization lags far behind the development of material civilization, especially the humanistic care for disadvantaged groups still needs considerable progress. Therefore, humanistic care has become the most crucial part of the current community building for aging. The overall satisfaction of the community's aging construction has an inverse linear relationship with the socio-economic dimension. This is because, limited by the lack of social elderly care resources and the shortage of pension funds, the current community-provided elderly care services such as housekeeping services, life care, intermediary assistance, and canteens for the elderly are not free. Thus, the higher the socio-economic aspects of aging constructions, the higher the cost in the community, and the lower the overall satisfaction with the aging construction (39). Therefore, enriching social home-based care resources and improving the level of social home-based care security is the future development direction of the community home-based care model, and the government faces problems that need to be resolved in the construction of an aging community.

### 5.2. Research suggestions

This study mainly aimed at constructing the evaluation index system of the aging construction of the community, and analyzed the influencing factors of overall satisfaction with the aging construction. It is suggested that the scope of research can be extended to urban or rural areas to understand the aging construction status in those areas. This will not only improve the index system for aging construction in society and contribute to a comprehensive strategy for elderly home-based care, but also further analyze the differences between the needs for aging construction among the elderly in urban and rural areas.

Community home-based care is a complex social issue involving many dimensions such as economic factor, medical factor, environmental factor, and social security. This paper discusses the evaluation index system of the aging construction of the community from the perspective of the elderly's caring needs, but there are still many shortcomings and directions for further research in the future.

The article studies the elderly as a group, aiming to discuss the needs of the elderly as a whole. However, the elderly are not a homogeneous group, and there are inevitably large differences in needs between regions and groups of elderly. Although the article notes the differences in the needs caused by the elderly's individual differences, it does not discuss them in depth, so in the future, further refinement may be made according to the differences in the needs of the elderly.

The evaluation index system of the aging construction of the community in this article were obtained through the previous research, but since the Chinese community home-based care model is in a period of rapid promotion and development, The standards of the aging construction of the community will certainly be further developed. Therefore, the uncertainty of future development is unavoidable in the research design of evaluation index system of the aging construction of the community.

This study has preliminarily established an evaluation index system of the aging construction of the community. The future research direction will use this as a basis for confirmatory analysis. The indicators and criteria of the index system will be quantified, and the weightage of indexes at all levels will be calculated to assess the community's aging suitability. On the one hand, it can evaluate the renewed community and suggest rectification suggestions. On the other hand, it can provide a decision-making basis for constructing the aging construction of an unrenewed community.

## Data availability statement

The original contributions presented in the study are included in the article/supplementary material, further inquiries can be directed to the corresponding author.

## Author contributions

Conceptualization and validation: KL and W-BM. Methodology, formal analysis, investigation, writing – original draft preparation, writing – review and editing, supervision, and project administration: KL. Software, resources, data curation, and funding acquisition: W-BM and Y-ZH. All authors have read and agreed to the published version of the manuscript.
